# Primary central nervous system lymphomas at a tertiary academic hospital in Johannesburg

**DOI:** 10.4102/jcmsa.v3i1.135

**Published:** 2025-07-04

**Authors:** Garrick E. Laudin, Vinitha Philip, Sugeshnee Pather, Atul Lakha, Romana Jassat, Faadil Waja, Ahshish Govind, Lindokuhle Goqwana, Moosa Patel

**Affiliations:** 1 Department of Clinical Haematology, Faculty of Health Sciences, School of Clinical Medicine, University of the Witwatersrand, Johannesburg, South Africa; 2 Department of Anatomical Pathology, Faculty of Health Sciences, School of Pathology, University of the Witwatersrand, Johannesburg, South Africa; 3 Department of Anatomical Pathology, National Health Laboratory Service, Chris Hani Baragwanath Academic Hospital, Johannesburg, South Africa; 4 Department of Internal Medicine, Faculty of Health Sciences, University of the Witwatersrand, Johannesburg, South Africa; 5 Department of Neurology, Faculty of Health Sciences, School of Clinical Medicine, University of the Witwatersrand, Johannesburg, South Africa

**Keywords:** Human immunodeficiency virus, non-Hodgkin lymphoma, large B-cell lymphomas, immune-privileged sites, central nervous system

## Abstract

**Background:**

Primary central nervous system lymphomas (PCNSL) are diffuse large B-cell lymphomas (DLBCL), which affect multiple regions of the neuroaxis, including the brain parenchyma, the orbits, cranial nerves and leptomeninges, without systemic disease. The aim of this study was to review the clinical characteristics as well as the outcome of patients referred to a tertiary academic hospital in Johannesburg (South Africa) with a presumed PCNSL diagnosis based on histology, cerebrospinal fluid (CSF) analysis and neuroimaging.

**Methods:**

This retrospective record review at a tertiary academic hospital in Soweto (Johannesburg), included patients aged ≥ 17 years referred with a presumed diagnosis of PCNSL, based on biopsy-confirmed PCNSL, CSF analysis or neuroimaging, between January 2010 and December 2022. Patient characteristics (laboratory, clinical, treatment(s), outcome) were analysed in three groups based on diagnostic certainty for PCNSL (diagnostic, suspicious, consistent).

**Results:**

The cohort comprised 14 patients, with most cases occurring in People Living with Human Immunodeficiency Virus (PLWH) (*n* = 10; 71%), with equal representation of male and female patients. The median age of diagnosis was 43 years (17–64), with PLWH diagnosed at an earlier mean age of 40 compared to human immunodeficiency virus (HIV)-negative patients (mean age 52). Both surviving patients received between 6 and 8 cycles of high-dose methotrexate (HD-MTX), with one receiving an autologous stem cell transplant. Over half (57%) of the cohort died with a median overall survival of two months (95% CI 1.3–2.6).

**Conclusion:**

Establishing a PCNSL diagnosis without histology often relies on several ancillary investigations. The high mortality and poor median survival highlight the importance of timely diagnosis and treatment.

**Contribution:**

This case series highlights the the complexities of a PCNSL diagnosis in PLWH and will hopefully inspire future work in this area.

## Introduction

Primary central nervous system lymphomas (PCNSL) are diffuse large B-cell lymphomas (DLBCL), which affect multiple regions of the neuroaxis, including the brain parenchyma, orbits, cranial nerves and leptomeninges, without the presence of concomitant systemic disease.^1,2,3,4,5^ The original description of the disease by Bailey in 1929 was that of a ‘perivascular sarcoma’, owing to the histological observation of mass-forming large lymphocytes arranged around blood vessels.^5^ The 5th edition of the World Health Organization (WHO) and its classification of lymphoid tumours published in 2022 classifies primary lymphoid malignancies of the central nervous system (CNS) under the umbrella term of ‘large B-cell lymphomas (LBCL) of immune-privileged sites’.^5,6^ The immune sanctuaries of the central nervous system and orbit are unique and exist because of the structure of the blood–brain and blood–retinal barriers, respectively.^2^

Sub-Saharan Africa is at the epicentre of the human immunodeficiency virus (HIV) pandemic, with data from Statistics South Africa (StatsSA) noting that 8.2 million people were living with HIV (PLWH) in South Africa in 2021, with an estimated prevalence of 13.7% of the general population affected.^7^ The PCNSLs represent one of the four acquired immunodeficiency syndrome (AIDS)-defining malignancies in PLWH. Antiretroviral therapy (ARV) naive PLWH carry a 3600-to-5000-fold increased risk of developing PCNSL, compared with the risk in the general population.^4,5^ Patients with advanced age, iatrogenic immune suppression (solid organ transplant recipients), as well as those with autoimmune conditions, are other at-risk groups for developing PCNSL.^5^

Although the gold standard diagnosis for a PCNSL is histological, this descriptive review also analyses the clinical characteristics and outcomes of adult patients at a tertiary academic hospital in Johannesburg (South Africa), in whom diagnosis was established by cerebrospinal fluid (CSF) analysis and/or neuroimaging.^8^

## Research methods and design

Patients included in this retrospective record review at a tertiary academic hospital in Soweto (Johannesburg), were aged ≥ 17 years, and referred to our unit with a presumed diagnosis of PCNSL, based on CSF analysis (cytology, flow cytometry, immunoglobulin heavy chain gene rearrangement), neuroimaging and/or a biopsy-confirmed histological diagnosis of PCNSL, between 01 January 2010 and 31 December 2022.

A total of 16 patients were assessed for study inclusion, with 2 excluded because of incomplete information regarding computerised tomography (CT) findings and data on treatment administered. The 14 patients included in this study were stratified into three groups based on the diagnostic certainty for PCNSL: (1) diagnostic (biopsy with histology) – 9 patients; (2) highly suspicious (CSF monoclonal immunoglobulin heavy chain gene re-arrangement and neuroimaging) – 3 patients; and (3) suspicious (neuroimaging alone) – 2 patients.

Patients’ characteristics (laboratory, clinical, treatment, outcome) were analysed and compared in each diagnostic certainty group. Kaplan-Meier analyses were used for patient survival, with quantitative variables expressed as mean or median and range.

Access to medical records at our institution was granted by the heads of the unit and the hospital medical advisory committee (MAC).

### Ethical considerations

Ethical clearance to conduct this study was obtained from the University of the Witwatersrand Human Research Ethics Committee (Medical) (No. M220941).

## Results

Among the 14 patients, the majority (*n* = 10; 71%) were PLWH (Table 1). The mean and median patient age was 43 years (range: 17–47) with equal male and female distribution. Compared with HIV-negative patients, PLWH were diagnosed with PCNSL at an earlier age (Table 1). Among PLWH (Table 2a, Table 2b, Table 2c), the mean cluster of differentiation (CD4) cell count was 107 (range: 33–226 cells/mm^3^) with HIV viral loads (HIVVL) in 3 patients (who defaulted ARVs) markedly elevated compared to the HIVVLs of 2 patients on ARVs (Table 2a, Table 2b, Table 2c). Half of PLWH were on fixed combination ARVs at the time of referral (duration of use unknown), 3 patients (30%) defaulted ARVs, and 2 patients (20%) had never been on ARVs (Table 1). Five patients (35%) were on anti-tuberculosis (anti-TB) treatment, 3 (21%) on treatment at referral, with empiric anti-TB therapy commenced in 2 (14%) patients. Of the 5 patients on anti-TB treatment, more than half (*n* = 3; 60%) had a detectable HIVVL more than 1000 copies/mL (Table 2a, Table 2b, Table 2c).

**TABLE 1:** Patients’ demographics and presenting symptoms: Diagnostic certainty for PCNSL diagnosis.

Parameter	Diagnostic (biopsy – brain or ocular)			Highly suspicious (CSF IgH monoclonal + imaging)			Suspicious (*Neuroimaging*)			Group total		
	*n*	*N*	%	*n*	*N*	%	*n*	*N*	%	*n*	*N*	%
**Demographics and patient characteristics**												
Number of patients	9	-	-	3	-	-	2	-	-	-	14	-
Male (*n*)	3	-	-	2	-	-	2	-	-	7	14	50
Female (*n*)	6	-	-	1	-	-	0	-	-	7	14	50
PLWH†	-	-	-	-	-	-	-	-	-	10	-	-
HIV neg‡	-	-	-	-	-	-	-	-	-	4	-	-
Race	-	-	-	-	-	-	-	-	-	14	-	-
Black people	8	9	89	3	3	100	1	2	50	12	14	86
Indian people	1	9	11	-	-	-	-	-	-	1	14	7
Coloured people (mixed)	-	-	-	-	-	-	1	2	50	1	14	7
HIV positive	5	9	55	3	3	-	2	2	-	10	14	71
PLWH and ARV use (*n* = 10)	-	-	-	-	-	-	-	-	-	-	-	-
ARV naive	1	5	20	-	-	33	1	2	50	2	10	20
On ARVs (duration unknown)	3	5	60	1	3	66	1	2	50	5	10	50
Defaulted ARVs	1	5	20	2	3	-	-	-	-	3	10	30
Anti-TB therapy (*n* = 14)	-	-	-	-	-	-	-	-	-	-	-	-
Anti-TB treatment at referral	1	9	11	1	3	33	1	2	50	3	14	21
Empiric anti-TB treatment	-	-	-	2	3	66	-	-	-	2	14	14
ECOG (PS) (*n* = 11)	-	-	-	-	-	-	-	-	-	-	-	-
≥ 4	5	7	71	2	3	66	1	1	100	8	11	73
< 4	2	7	29	1	3	33	-	-	-	3	11	27
**Presenting symptoms**												
B-symptoms (*n* = 10)	4	7	-	3	3	-	-	-	-	7	10	70
B-symptoms (+ concurrent TB treatment)	-	-	-	3	3	-	-	-	-	3	7	43
Visual loss	5	9	56	-	-	-	-	-	-	5	14	36
Altered LOC	-	-	-	2	3	66	2	2	100	4	14	29
Headache	1	9	11	1	3	33	-	-	-	2	14	14
Seizures	1	9	11	-	-	-	-	-	-	1	14	7
Weakness (focal)	1	9	11	-	-	-	-	-	-	1	14	7
Ataxia	1	9	11	-	-	-	-	-	-	1	14	7

*Source*: Table headings adapted from Scott B, Douglas V, Tihan T, Rubenstein J, Josephson S. A systematic approach to the diagnosis of suspected central nervous system lymphoma. JAMA Neurol. 2013;70(3):311. https://doi.org/10.1001/jamaneurol.2013.606

Note: Age (years): mean = 44; Diagnostic (biopsy – brain or ocular): median = 44; range: 17–64. Highly suspicious (CSF IgH monoclonal + imaging): median = 42; range: 36–47. Suspicious (*Neuroimaging*): median = 42; range: 40–44. Group total: median = 43; range: 17–64.

ARV, anti-retroviral therapy; ECOG, eastern co-operative oncology group; LOC, level of consciousness; PS, performance status; PCNSL, primary central nervous system lymphoma; PLWH, people living with human immunodeficiency virus; TB, tuberculosis.

† , PLWH: median = 40; range: 17–47;

‡ , HIV negative: median = 52; range: 23–64.

**TABLE 2a:** Histological, laboratory and neuroimaging results: Diagnostic categories pf PCNSL.

Parameter	Diagnostic (biopsy – brain or ocular)			Highly suspicious (CSF IgH monoclonal + imaging)		Suspicious (*Neuroimaging*)		Group total		
	*n*	*N*	%	Description	%	Description	%	*n*	*N*	%
**Histology**										
NHL - DLBCL	8	9	89	No histology: diagnosis **highly suspicious** for PCNSL based on CSF monoclonal IgH and neuroimaging	-	No histology: diagnosis based on neuroimaging findings **consistent** with PCNSL	-	8	9	89
NHL - Burkitt lymphoma	1	9	11					1	9	11

*Source:* Table headings adapted from Scott B, Douglas V, Tihan T, Rubenstein J, Josephson S. A systematic approach to the diagnosis of suspected central nervous system lymphoma. JAMA Neurol. 2013;70(3):311. https://doi.org/10.1001/jamaneurol.2013.606

NHL, non-Hodgkin lymphoma; DLBCL, diffuse large B-cell lymphoma; CSF, cerebrospinal fluid; IgH, immunoglobulin heavy chain gene; PCNSL, primary central nervous system lymphoma.

**TABLE 2b:** Histological, laboratory and neuroimaging results: Diagnostic categories pf PCNSL.

Parameter	Diagnostic (biopsy – brain or ocular)		Highly suspicious (CSF IgH monoclonal + imaging)†		Suspicious (*Neuroimaging*)		Group total‡		
	Mean	Range	Mean	Range	Mean	Range	Mean	Range	Median
**Laboratory investigations**									
WCC (x10^9/L) (RI = 3.9 – 12.60)	6.5	3.63–9.97	3.93	-	6.56	-	6.30	3.63–9.97	5.64
Hb (g/dl) (RI = 11.6 - 16.4)	11.62	9.30–13.50	10.47	10–11.40	11.95	9.70–14.20	11.42	9.30–14.50	11.35
Platelets (x 10^9/L) (RI = 186 – 454)	346	213–49	268	234–297	246	204–289	315	204–495	293
LDH (μ/L) (RI = 100 – 190)	321	155–430	214	191–237	739	-	339	155–739	298
CD4 cell count (cells/μL)	127	33–226	120	71–189	40	35–46	107	33–226	91
HIV viral load (copies/mL)	250044	24–1000000	47983	1500–94466	-	-	182690	24–100000	-
HIV VL (defaulted ARV, 3 pts)	-	-	-	-	-	-	365322	-	-
HIV VL (on ARVS, 2 pts)	-	-	-	-	-	-	32	-	-
HIV VL (unclear of Rx, 1 pt)	-	-	-	-	-	-	114	-	-
HIV VL > 1000 and on anti-TB treatment	1 patient	-	2 patients	-	-	-	-	-	-

*Source:* Table headings adapted from Scott B, Douglas V, Tihan T, Rubenstein J, Josephson S. A systematic approach to the diagnosis of suspected central nervous system lymphoma. JAMA Neurol. 2013;70(3):311. https://doi.org/10.1001/jamaneurol.2013.606

WCC, white cell count; RI, reference interval; HIV VL, human immunodeficiency virus viral load; LDH, lactate dehydrogenase; ARV, anti-retroviral therapy; TB, tuberculosis; PCNSL, primary central nervous system lymphoma.

† , Toxoplasmosis IgG positive: 1/3 (33%);

‡ , Toxoplasmosis IgG positive: 1/3.

**TABLE 2c:** Histological, laboratory and neuroimaging results: Diagnostic categories pf PCNSL.

Parameter	Diagnostic (biopsy – brain or ocular)			Highly suspicious (CSF IgH monoclonal + imaging)			Suspicious (*Neuroimaging*)			Group total		
	*n*	*N*	%	*n*	*N*	%	*n*	*N*	%	*n*	*N*	%
**CSF analysis**												
Pleocytosis (≥ 5 leucocytes/μL)	3	5	60	2	3	66	2	2	100	7	10	70
IgH monoclonal band	1	2	50	3	3	100	-	-	-	3	3	100
**Imaging**												
Lesion (number) (*n* = 14)	6	9	66	1	3	33	2	2	100	7	14	50
Single	3	9	33	2	3	66	2	2	100	7	14	50
Multiple (> 1)	6	9	-	2	3	67	-	-	-	10	14	71
Lesion (site) (*n* = 14)	2	9	-	-	-	33	-	-	-	2	14	14
Deep white matter	1	9	-	-	-	-	-	-	-	1	14	7
Ocular apparatus	-	-	-	-	-	-	-	-	-	1	14	7
Cerebellum	-	-	-	-	-	-	-	-	-	-	-	-
Brain stem	-	-	-	1	3	-	-	-	-	-	-	-
Cerebral venous sinus thrombosis	2	9	-	-	-	-	-	-	-	2	14	1.43

*Source:* Table headings adapted from Scott B, Douglas V, Tihan T, Rubenstein J, Josephson S. A systematic approach to the diagnosis of suspected central nervous system lymphoma. JAMA Neurol. 2013;70(3):311. https://doi.org/10.1001/jamaneurol.2013.606

Note: Protein: *n* = 9; Diagnostic (biopsy – brain or ocular): mean = 0.62; range: 0.25–0.99. Highly suspicious (CSF IgH monoclonal + imaging): mean = 0.75; range: 0.37–1.25. Suspicious (*Neuroimaging*): mean = 1.1; range: 0.53–1.7. Group total: mean = 0.7; range: 0.25–1.25.

CSF, cerebrospinal fluid; IgH, immunoglobulin heavy chain gene; PCNSL, primary central nervous system lymphoma.

Presenting symptoms included visual loss (*n* = 5; 36%), an altered level of consciousness (LOC) (*n* = 4; 29%), headache (*n* = 2; 14%), seizures (*n* = 1; 7%), focal weakness (*n* = 1; 7%) and ataxia (*n* = 1; 7%) (Table 1). Eight patients (73%) in whom performance status was recorded, according to the Eastern Co-operative Oncology Group (ECOG) classification, were ECOG grade four (Karnofsky equivalent score of 20). The presence of B-symptoms was documented in 10 of 14 patients in this cohort. Of these 10 patients, 7 had at least one B-symptom (70%), and 3 of the 7 (43%) were also receiving concurrent anti-TB treatment (Table 1).

**FIGURE 1:**
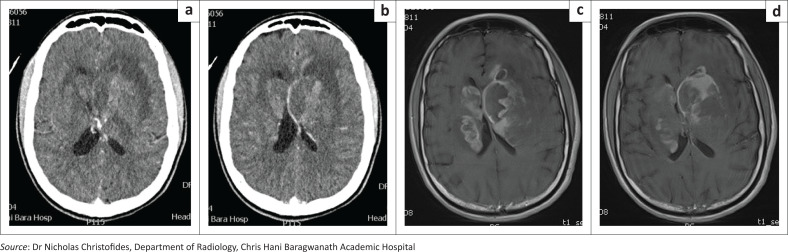
Typical CT scan findings from a patient in this case series. (a) Selected axial slices of contrast-enhanced CT brain displaying a heterogeneous midline lesion involving bilateral cssaudate nuclei and corpus callosum with associated adjacent vasogenic oedema; (b) Selected axial slices of T1-weighted contrast-enhanced magnetic resonance imaging (MRI) brain confirming the midline lesion with invasion of bilateral basal ganglia, caudate nuclei and corpus callosum. The lesion displays heterogeneous peripheral enhancement and perilesional oedema. There is no associated dural enhancement to suggest dural spread.

Enumeration of CNS lesions was equally split with 7 (50%) patients with a single CNS lesion and 7 (50%) with multiple lesions. Most patients with more than 1 CNS lesion were PLWH (6/7 patients; 85%). Lesions of the CNS were localised to deep structures of the brain parenchyma in the majority (*n* = 10; 71%), the ocular apparatus in 6% of patients (*n* = 2), with brainstem and cerebellum involved in 2 patients, respectively. Typical CT scan findings of a patient in this cohort are noted in Figure 1.

Histological analysis of brain and ocular biopsy specimens was classified as diagnostic for PCNSL in 9 patients, 5 of which were obtained by craniotomy, 2 from ocular mass biopsy and 2 from stereotactic brain biopsies. All 9 histological specimens were B-cell non-Hodgkin lymphoma (NHL), DLBCL in the majority (*n* = 8; 88%) and Burkitt lymphoma in 1 patient (Table 1). In 1 patient with DLBCL and undifferentiated connective tissue disease, the Epstein–Barr encoding region *in situ* hybridisation (EBER-ISH) was performed and was found to be positive.

The baseline laboratory characteristics of the 14 patients are included in Table 1. Serum toxoplasmosis serology was available for 4 patients, 3 of whom were negative (immunoglobulin M [IgM] and immunoglobulin G [IgG] were negative), with IgG serology positive in 1 patient, indicating past infection.

Five patients (42%) received an average 2 cycles of standard dose cyclophosphamide, hydroxydaunorubicin, vincristine sulfate and prednisone (CHOP), as their first course of chemotherapy, with 2 patients receiving a second course of chemotherapy with 1–2 cycles of high-dose methotrexate (HD-MTX) (Table 3). One patient received only corticosteroids with complete remission of the lesion on follow-up radiographic studies. Five patients (42%) received an HD-MTX-based chemotherapy and 3 patients with the addition of cytosine arabinoside to HD-MTX, as the first course of treatment. Both surviving patients in this cohort received 6–8 cycles of an HD-MTX-based regimen. Four patients received intrathecal chemotherapy (IT). Three patients received whole brain radiation therapy (WBRT), of whom 2 patients are still alive.

**TABLE 3:** Patient treatment, outcome and cause of death: Diagnostic categories of PCNSL.

Parameter	Diagnostic (biopsy – brain or ocular)			Highly suspicious (CSF IgH monoclonal + imaging)			Suspicious (*Neuroimaging*)			Group total		
	*n*	*N*	%	*n*	*N*	%	*n*	*N*	%	*n*	*N*	%
**Treatment**												
CHOP-based first line	2	9	22	1	3	33	2	2	100	5	14	36
HD-MTX-based first line	4	9	44	1	3	33	-	-	-	5	14	36
Corticosteroids only	-	-	-	1	3	33	-	-	-	1	14	7
Demised prior to chemo	1	9	11	-	-	-	-	-	-	1	14	7
Treatment unknown	2	9	22	-	-	-	-	-	-	2	14	14
Whole brain radiation therapy (WBRT)	2	9	22	0	3	-	1	2	50	3	14	21
Autologous stem cell transplant	1	9	11	0	3	-	0	2	-	1	14	7
Intrathecal (IT) chemotherapy	1	9	11	2	3	66	0	2	-	3	14	21
**Outcome**												
Alive	2	9	22	-	-	-	-	-	-	2	14	14
Demised	4	9	44	2	3	66	2	2	-	8	14	57
Unknown	3	9	33	1	3	-	-	-	-	4	14	29
**Cause of death**												
Sepsis and/or infection	2	4	50	-	-	-	1	2	50	3	8	37
Advanced disease (PCNSL)	2	4	50	-	-	-	-	-	-	2	8	25
Advanced HIV infection	-	-	-	1	2	50	-	-	-	1	8	13
Unknown	-	-	-	1	2	50	1	2	50	2	8	25

*Source*: Table headings adapted from Scott B, Douglas V, Tihan T, Rubenstein J, Josephson S. A systematic approach to the diagnosis of suspected central nervous system lymphoma. JAMA Neurol. 2013;70(3):311. https://doi.org/10.1001/jamaneurol.2013.606

Note: Survival (days): Diagnostic (biopsy – brain or ocular): mean = 85; range: 27–147. Highly suspicious (CSF IgH monoclonal + imaging): mean = 110; range: 49–172. Suspicious (Neuroimaging): mean = 60; range: 53–67. Group total: mean = 85; range: 27–172. Survival (months): Diagnostic (biopsy – brain or ocular): median = 2. Highly suspicious (CSF IgH monoclonal + imaging): median = 1.6. Suspicious (Neuroimaging): median = 1.7. Group total: median = 2; range: 1.3–2.6.

CHOP, cyclophosphamide, hydroxydaunorubicin, vincristine sulphate/oncovin and prednisone; HD-MTX, high-dose methotrexate; IT, intrathecal chemotherapy; WBRT, whole brain radiation therapy; PCNSL, primary central nervous system lymphoma; CSF, cerebrospinal fluid; IgH, immunoglobulin heavy chain gene.

Over half of the patients in the cohort demised (*n* = 8; 57%), 2 patients (14%) in our series alive with 5 patients (31%) lost to follow up. The cause of death in 8 patients was attributed to sepsis and/or infection (*n* = 3; 37%), advanced central nervous system disease (*n* = 2; 25%) and sequelae of poorly controlled HIV infection (*n* = 1; 13%). Five patients were lost to follow up and their outcome is unknown.

**FIGURE 2:**
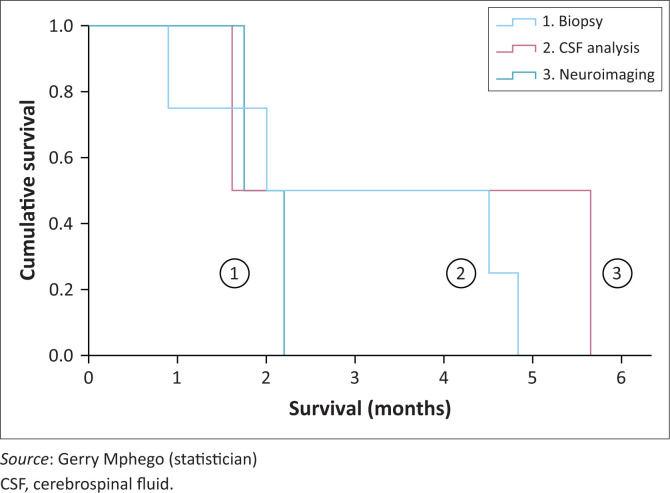
Survival analysis in three diagnostic patient subgroups.

The median overall survival for the cohort was two months (95% CI 1.3–2.6), with the difference between the distributions of median overall survival in each of the three patient diagnostic subgroups (diagnostic, highly suspicious and suspicious) not statistically significant (*p* > 0.05) (Figure 2).

## Discussion

In this retrospective chart review, we report on a cohort of patients diagnosed with PCNSL based on: (1) a tissue biopsy of CNS lesions – regarded as the gold standard for diagnosis; (2) CSF pleocytosis (> 5 leucocytes per µL) with a monoclonal IgH band – ‘highly suspicious’ for PCNSL; and (3) neuroimaging (CT and/or MRI) findings.^8^ The different methods of diagnosis are reflective of the discipline (surgical or medical) to which the patient initially presents. Surgical disciplines, particularly neurosurgery, use tissue-based methods as a means of diagnosis, as non-lymphomatous CNS lesions would be higher on the list of differentials. When an immunocompromised patient, either because of HIV or chronic immunosuppression, presents to a medical discipline, the differential diagnosis and diagnostic algorithm largely favour an infectious aetiology.

The differential diagnosis of intracranial mass lesions in PLWH is broad, with the incidence of opportunistic infection (Mycobacterium tuberculosis, toxoplasmosis, *Cryptococcus*) inversely associated with CD4 T-lymphocyte count. Tuberculosis is a leading cause of mortality in PLWH, and anti-TB therapy (together with corticosteroids) is often started empirically in critically ill hospitalised patients, which is particularly true of patients with undiagnosed intracranial mass lesions.^9^ Cerebral toxoplasmosis is an important differential for CNS disease in PLWH, however the frequency of serological testing in our cohort was low. Although the exact prevalence of toxoplasmosis in South Africa is unknown, serological testing should be considered in PLWH (ARV naive) with CD4 cell counts less than 50 cells/µL.^10^

The incidence of PCNSL is inversely correlated with CD4 cell count, with a notable decline in the incidence in PLWH treated with combination ARV.^11,12^ The mean CD4 T-lymphocyte count in 10 of the 14 HIV-positive patients in our series was 107 cells/µL, with a median of 91.5 cells/µL. The PCNSL develops in patients with more severe immunodeficiency, usually at a median CD4 of 10 cells/µL, compared with patients with systemic NHL, in which disease develops at a median CD4 count of 189 cells/µL.^13^

The mean HIVVL was markedly higher in PLWH who defaulted ARVS, underscoring uncontrolled HIV in PCNSL pathogenesis.^14^ Incomplete viral suppression may have predictive value in the long-term risk of tumorigenesis, particularly in those malignancies associated with chronic inflammation and viral coinfection.^14^ It is therefore not surprising that cited favourable prognostic factors in HIV positive cohorts include a CD4 count greater than 200 cells/μL and an HIV viral load less than 400 copies/mL.^11^ Other prognostic markers in patients with PCNSL include a raised serum beta-2-microglobulin level (≥ 1.8 μg/mL). Although B2MG levels were increased in this cohort, future studies will be required to assess the significance in PCNSL and perhaps PLWH.^15^

Advanced age is a further risk factor for PCNSL as the disease occurs with increasing frequency in patients in their 6th to 7th decade of life.^11,16^ Patients aged > 60 account for more than half of all PCNSL cases and up to 20% of all patients with PCNSL are > 80 years of age. The median age of a PCNSL diagnosis in literature is approximately 67 years of age.^5^ In this cohort, patients were diagnosed at a mean and median age of 43 years of age, with the youngest patient diagnosed at 17 years of age. The difference in the median age of onset recorded in published data from that in our cohort, is likely reflective of the relative risk that HIV-related immunodeficiency plays in the earlier development of PCNSL, with an increase in PCNSL in non-HIV positive patients seen more with advancing age.^1,4^

Although current guidelines endorse high-dose methotrexate (HD-MTX) as the chemotherapeutic agent of choice, 6 patients in our cohort received standard CHOP as their first course of chemo-immunotherapy.^17^ The CHOP and the addition of rituximab to standard CHOP (R-CHOP) are generally considered in the treatment of systemic diffuse large B-cell NHL, and not as therapeutic options in PCNSL.^18^ The main concern in the treatment of CNS disease is the drug’s ability to cross the blood–brain barrier (BBB), with agents used in R-CHOP unable to cross the BBB.^11,19^ The HD-MTX is the preferred agent of choice, as cytotoxic levels are generally achieved within the CSF following intravenous (IV) administration of methotrexate at doses greater than 3 g per square meter.^18^ Moreover, HD-MTX is considered a relatively safe treatment for patients with PCNSL, regardless of age, and is tolerable in patients aged > 80 years. While 4 patients received intrathecal chemotherapy, the additional benefit of intrathecal therapy remains unclear given the ability of intravenous HD-MTX in achieving therapeutic concentrations within the CSF.^1^

Two patients in this series received multimodality therapy comprising chemotherapy and WBRT. The WBRT is associated with significant delayed neurotoxicity, particularly in patients over the age of 60.^1,17^ The 2 surviving patients in our cohort who received consolidative WBRT developed no overt neurotoxicity post-therapy. The WBRT is advantageous, as it provides more durable disease control, and in patients < 60 years of age results in improved survival and quality adjusted life years.^1^ Primary WBRT remains a treatment option for patients who remain unfit for HD-MTX.^11,16^

The observed high mortality rate of 57% (*n* = 8), and poor median overall survival (OS) of two months (95% CI 1.3–2.6), highlights a worse prognosis compared to larger cohort studies, likely reflective of diagnostic delays (limited neurosurgical access, inoperable brain lesions), advanced disease at presentation and suboptimal HIV control.^20,21,22^ The small number of patients in our study precludes robust statistical analysis, limiting the ability to detect significant differences in median OS across the three diagnostic subgroups (diagnostic, highly suspicious and suspicious). Consequently, the generalisability of our study’s findings is constrained because of restricted statistical power.

## Conclusion

The PCNSL remains an important consideration in PLWH, particularly those with advanced disease or detectable HIVVLs. Obtaining histological specimens for PCNSL diagnosis is challenging owing to several patient or disease-related factors and diagnosis may rely on a composite of characteristic imaging findings, CSF analysis (pleocytosis, monoclonal band) and exclusion of other important differentials in PLWH. With a notably poor survival in our cohort, optimal management of patients with PCNSL is dependent on early disease diagnosis, optimisation of patient performance status and access to chemoimmunotherapy.

Statistics on PCNSL incidence in the South African setting remain limited and represent a focus for future work in this area.
